# Evolution avoids a pathological stabilizing interaction in the immune protein S100A9

**DOI:** 10.1073/pnas.2208029119

**Published:** 2022-10-04

**Authors:** Joseph L. Harman, Patrick N. Reardon, Shawn M. Costello, Gus D. Warren, Sophia R. Phillips, Patrick J. Connor, Susan Marqusee, Michael J. Harms

**Affiliations:** ^a^Department of Chemistry and Biochemistry, University of Oregon, Eugene, OR 97403;; ^b^Institute of Molecular Biology, University of Oregon, Eugene, OR 97403;; ^c^College of Science, NMR Facility, Oregon State University, Corvallis, OR 97331;; ^d^Biophysics Graduate Program, University of California, Berkeley, Berkeley, CA 94720;; ^e^Department of Molecular and Cell Biology, University of California, Berkeley, Berkeley, CA 94720;; ^f^Department of Chemistry, University of California, Berkeley, Berkeley, CA 94720;; ^g^California Institute for Quantitative Biosciences, University of California, Berkeley, Berkeley, CA 94720

**Keywords:** protein evolution, protein stability, protein folding, mutation, innate immune system

## Abstract

Stability constrains evolution. While much is known about constraints on destabilizing mutations, less is known about the constraints on stabilizing mutations. We recently identified a mutation in the innate immune protein S100A9 that provides insight into such constraints. When introduced into human S100A9, M63F simultaneously increases the stability of the protein and disrupts its natural ability to activate Toll-like receptor 4. Using chemical denaturation, we found that M63F stabilizes a calcium-bound conformation of hS100A9. We then used NMR to solve the structure of the mutant protein, revealing that the mutation distorts the hydrophobic binding surface of hS100A9, explaining its deleterious effect on function. Hydrogen–deuterium exchange (HDX) experiments revealed stabilization of the region around M63F in the structure, notably Phe37. In the structure of the M63F mutant, the Phe37 and Phe63 sidechains are in contact, plausibly forming an edge-face π-stack. Mutating Phe37 to Leu abolished the stabilizing effect of M63F as probed by both chemical denaturation and HDX. It also restored the biological activity of S100A9 disrupted by M63F. These findings reveal that Phe63 creates a molecular staple with Phe37 that stabilizes a nonfunctional conformation of the protein, thus disrupting function. Using a bioinformatic analysis, we found that S100A9 proteins from different organisms rarely have Phe at both positions 37 and 63, suggesting that avoiding a pathological stabilizing interaction indeed constrains S100A9 evolution. This work highlights an important evolutionary constraint on stabilizing mutations, namely, that they must avoid inappropriately stabilizing nonfunctional protein conformations.

## Significance

Protein stability constrains protein evolution. While much is known about evolutionary constraints on destabilizing mutations, less is known about the constraints on stabilizing mutations. We recently found a mutation to an innate immune protein that increases its stability but disrupts its function. Here, we show, through careful biophysical and functional studies, that this mutation stabilizes a nonfunctional form of the protein through a direct interaction with another amino acid. We find that specific amino acids can be tolerated at each of the interacting positions individually but not at both simultaneously. This pattern has been conserved over millions of years of evolution. We highlight an underappreciated evolutionary constraint on stabilizing mutations, namely, that they must avoid inappropriately stabilizing nonfunctional protein conformations.

Protein stability constrains protein evolution (1–5). This is intuitive for destabilizing mutations, as a mutation that disrupts the folded form of a protein will likely be deleterious. The evolutionary constraints on stabilizing mutations are, however, harder to understand. Under what circumstances does it matter that a mutation makes a protein too stable? Conflicting arguments have been advanced for enzyme stability–function tradeoffs. In many cases, increasing stability can decrease important dynamics related to catalysis or product release (6–9); in contrast, other studies show a positive correlation between enzyme stability and function (10, 11). The generic constraints on excess stability as proteins evolve, particularly for nonenzymatic proteins, remain poorly understood.

**Fig. 1.**
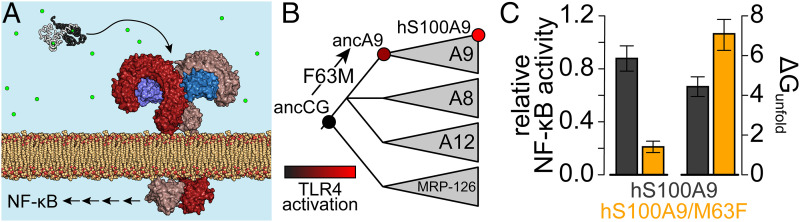
Reverting M63F in human S100A9 stabilizes the protein and disrupts proinflammatory activity. (*A*) Schematic showing a calcium-bound S100A9 dimer (white/black; PDB 1IRJ) in the calcium-rich extracellular space. S100A9 triggers dimerization of the TLR4 complex (red/blue; PDB 3FXI (68)) on the surface of immune cells, activating NF-κB and other pathways. (*B*) Schematic phylogeny of S100A9 and its close paralogs S100A8, S100A12, and MRP-126. Wedges denote paralogs from mammals (A9, A8, and A12) or sauropsids (MRP-126). We previously characterized the indicated nodes (ancCG, ancA9, hS100A9) (12, 17). The color of each node represents its relative ability to activate TLR4. The F63M mutation between ancCG and ancA9 was important for the evolution of S100A9’s proinflammatory activity. (*C*) Reverting M63F in hS100A9 compromises its ability to activate NF-κB via TLR4 (left), while increasing unfolding free energy in 5 mM Ca^2+^ (*Right*; experimental data shown in Fig. 2).

We recently identified a site in a protein that seemingly evolved to avoid deleterious stabilization (12), providing an excellent opportunity to probe how the requirement to avoid stabilizing mutations might constrain evolution. We isolated the mutation in question while studying the evolution of proinflammatory activity in mammalian S100A9 proteins. S100 proteins across amniotes activate the receptor Toll-like receptor 4 (TLR4) by an as-yet poorly understood mechanism (Fig. 1*A*) (13–16). This activity evolved in the ancestor of all mammals when a gene shared by all amniotes (ancCG) duplicated and diverged into the last common S100A9 ancestor (ancA9) (Fig. 1*B*) (12, 17). Using Ancestral Sequence Reconstruction (ASR), we found that ancCG had negligible proinflammatory activity, ancA9 had moderate activity, and S100A9 proteins from placental mammals had high activity (Fig. 1*B*).

This proinflammatory function arose, in part, when Met replaced Phe at position 63 in S100A9. Here, and throughout the manuscript, we identify amino acids by their numbering in the human sequence. Intriguingly, we found that reverting this position in human S100A9—hS100A9/M63F—both compromised the proinflammatory activity of the protein and increased its stability (Fig. 1*C*) (12). Relative to hS100A9, hS100A9/M63F required a higher concentration of urea to unfold, exhibited slower unfolding kinetics, and was much more resistant to proteolysis (12). We also showed that intact hS100A9, but not its proteolytic products, was the active TLR4 agonist, indicating that the mutation disrupts activity by altering the native functional state of the protein.

Here, we combine chemical denaturation experiments, structural biology, and hydrogen–deuterium exchange (HDX) to understand the molecular mechanism for the stabilizing and functionally disruptive effects of this mutation. We find that a single position, Phe37, forms a “molecular staple” with Phe63. This interaction stabilizes a distorted, nonfunctional structure of the protein. A bioinformatic analysis reveals that S100A9 proteins frequently have Phe at either site but rarely at both sites. Thus, for this family of immune proteins, evolution must avoid a pathological stabilizing interaction that compromises function by stabilizing nonfunctional protein conformations.

## Results

In our previous work, we found that reverting Met63 to its ancestral Phe state in human S100A9 (hS100A9) both increases its stability and decreases its proinflammatory activity (Fig. 1*C*) (12). To better understand evolutionary constraints on stability, we set out to understand the origins of this altered stability and function.

### M63F Stabilizes a Calcium-Bound Conformation of the Protein.

**Fig. 2.**
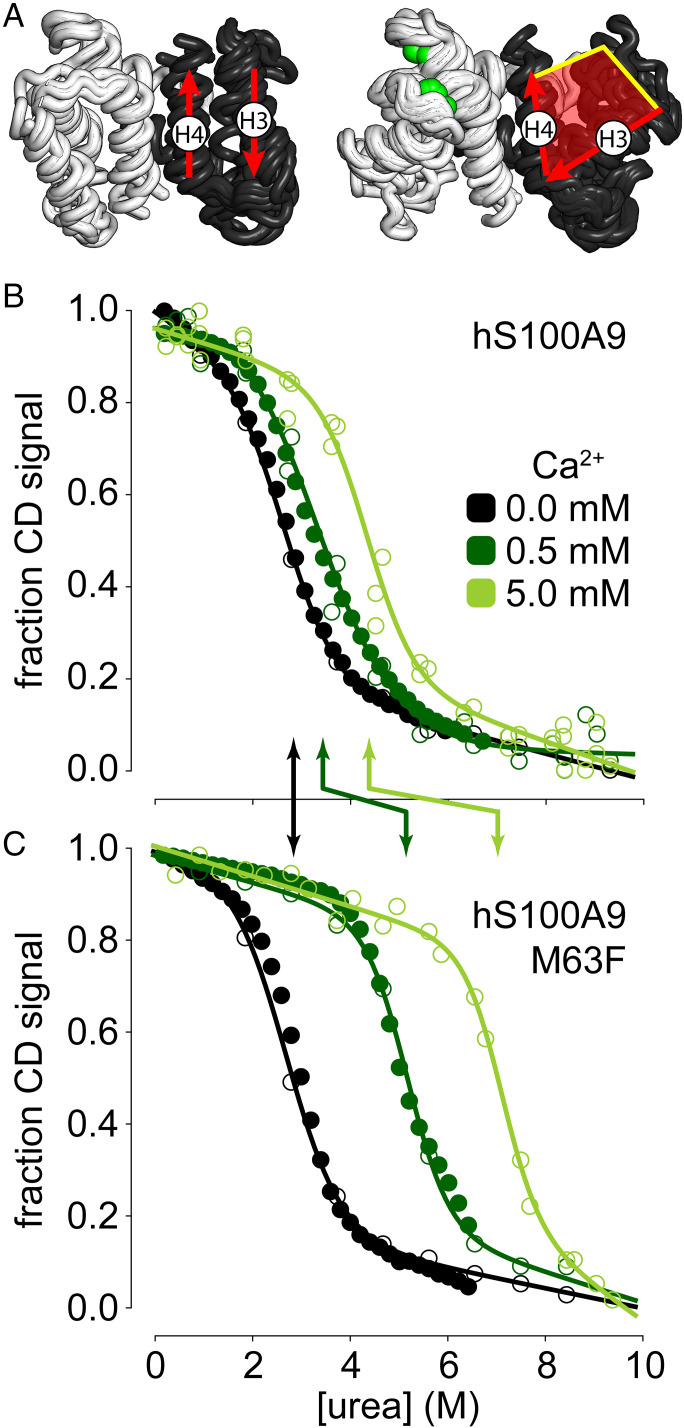
M63F stabilizes a calcium-bound form of hS100A9. (*A*) Overlay of the structures of multiple S100 paralogs in the apo (*n* = 7; left) and calcium-bound (*n* = 25; right) forms. Materials and Methods lists PDB identifiers. Only residues corresponding to residues 5 to 85 of hS100A9 are shown; dimer chains are shown in white and black; calcium is shown as green spheres. The red arrows indicate the average orientation of helix 3 and helix 4. The red shaded area for the calcium-bound structure indicates the exposed hydrophobic surface. (*B*) Urea denaturation of hS100A9 in 0 (black), 0.5 (dark green), and 5 mM (light green) Ca^2+^. The *y* axis is CD signal at 222 nm, normalized from 0 to 1. Closed circles are automated titrations; open circles are manual titrations. Lines are the best fit model to all experiments for that calcium condition. (*C*) Denaturation of hS100A9/M63F. Colors and symbols are as in *B*. The arrows between *B* and *C* indicate the midpoint of denaturation (C_m_, in M) for the two proteins.

We first hypothesized that the mutation disrupts function by stabilizing an inactive, calcium-free form of the protein. Like most S100 proteins, S100A9 forms a homodimer with two pairs of calcium binding sites (18). Upon calcium binding, S100A9 undergoes a conformational change that exposes a hydrophobic cleft (Fig. 2*A*; shaded red) (18). This cleft is the surface through which S100 proteins typically interact with target proteins (19–23). If the mutation stabilized an inactive, calcium-free conformation, this would explain how increased stability altered protein function.

To test this hypothesis, we determined which conformation(s) of hS100A9 were stabilized by M63F. We measured the unfolding free energy (ΔG_unfold_) of hS100A9 and hS100A9/M63F in increasing concentrations of calcium, as follows: 0 mM, 0.5 mM, and 5 mM Ca^2+^. We measured equilibrium unfolding of the protein in urea by circular dichroism (CD) spectroscopy monitored at 222 nm, which reports on protein α-helical content. We fit a two-state model to the data, which had free parameters describing ΔG_unfold_ in water, the dependence of ΔGunfold on urea concentration (m-value), and the native and denatured baselines (24). We analyzed the data in two ways. First, we fit a global m-value for all protein variant/calcium concentrations. This is a reasonable assumption for hS100A9 and hS100A9/M63F, as the proteins differ by a single amino acid and the m-value is strongly correlated with the amount of hydrophobic surface area that is exposed upon protein unfolding (25). These are the data presented throughout the text and figures. Second, we allowed the m-value to vary for each protein/calcium condition. The two approaches gave qualitatively similar outcomes (*SI Appendix*, Fig. S1 and Tables S1 and S2); however, we note where they diverge in the text.

In the absence of calcium, we observed no significant difference in apparent stability between hS100A9 and hS100A9/M63F, as follows: 2.6 ± 0.5 vs. 2.4 ± 0.5 kcal/mol (Fig. 2 *B* and *C*). Adding calcium stabilizes both hS100A9 and hS100A9/M63F, as expected from Le Chatelier’s principle. The effect was, however, much more dramatic for the M63F mutant than hS100A9. At 5 mM Ca^2+^, hS100A9 and hS100A9/M63F have stabilities of 4.3 ± 0.5 vs. 7.0 ± 0.5 kcal/mol, respectively. This can be seen visually by the much larger calcium-induced increase in urea melting midpoint for the mutant compared to the wild-type protein (Fig. 2 *B* and *C*; *SI Appendix*, Table S1 for fit parameters).

Because the mutation had no effect in the absence of calcium but exhibits an increasing effect size with increasing calcium, we concluded that the mutation preferentially stabilizes a calcium-bound form of the protein.

### M63F Distorts the Structure of hS100A9.

It was surprising that a mutation compromised activity while stabilizing a calcium-bound form of hS100A9. hS100A9 activates TLR4 in the extracellular space, where the concentration of calcium is two orders of magnitude above the K_D_ of binding for hS100A9 to calcium (26) (Fig. 1*A*); therefore, the calcium-bound state is presumably the active form of the protein (13–15). Further, for most members of the S100 protein family, the calcium-bound conformation preferentially interacts with downstream target proteins (19–22). How could stabilization of a calcium-bound state disrupt activity?

To answer this question, we solved the structure of the calcium-bound form of hS100A9/M63F in the presence of 10 mM CaCl_2_ by NMR. We transferred and confirmed peak assignments for hS100A9/M63F from the calcium-loaded wild-type hS100A9 NMR structure (27) using three-dimensional (3D) NMR experiments and successfully assigned peaks for 91 out of 114 residues in hS100A9/M63F (*SI Appendix*, Fig. S2). The majority of unassigned peaks are within the S100A9 disordered C-terminal tail, most of which are also unassigned in the wild-type hS100A9 structure (27).

**Fig. 3.**
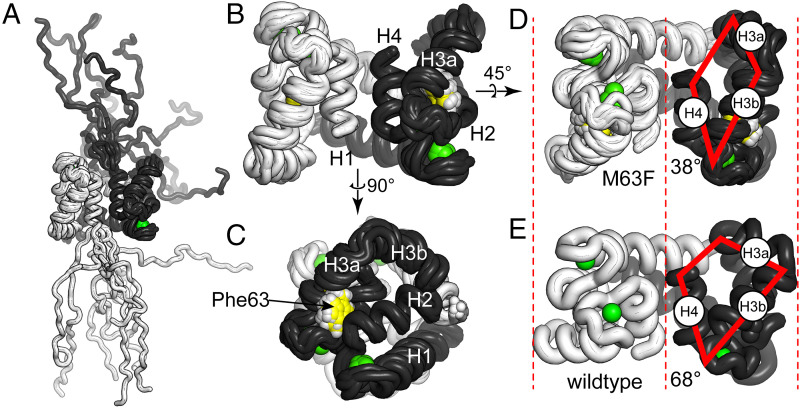
M63F distorts the structure of hS100A9. (*A*) Overlay of 10 models from the NMR ensemble generated for hS100A9/M63F. The A and B chains are shown as white and black, and calcium ions are shown in green. Residues 1 to 5 and 87 to 114 are poorly constrained in the structure. (*B* and *C*) Models for residues 6 to 86. *C* is rotated 90° relative to *B*. Helices 1, 2, 3a, 3b, and 4 are indicated on the structure. Phe63 is highlighted in yellow. (*D*) Structure shown in *B*, rotated 45° along indicated axis. The solid red lines highlight the S100A9 binding surface (as in Fig. 2*A*). (*E*) Crystal structure of hS100A9 (PDB ID: 1IRJ) (18) in the same orientation as the structure in *D*. The solid red lines highlight the hydrophobic binding cleft. The red dashed lines highlight the increased compaction of hS100A9/M63F relative to hS100A9.

The resulting ensemble of structures has a core set of residues (6 to 86) with an average pairwise backbone heavy atom rmsd of 0.9 Å. Detailed structural statistics are available in Table 1. The remaining residues (1 to 5 and 87 to 114) are poorly constrained by the NMR data (Fig. 3*A*). This is consistent with previous work showing that the N and C termini of hS100A9 are disordered, both in solution (27) and in a crystal structure of the protein (18). The well-ordered region of the protein adopts the same basic fold as observed in the crystal structure of wild-type hS100A9 (Fig. 3*B*). The average pairwise backbone heavy atom rmsd between the hS100A9/M63F NMR ensemble and the hS100A9 crystal structure (Protein Data Bank [PDB] identifier [ID]: 1IRJ) is 2.45 ± 0.2 Å.

**Table 1.** NMR structure statistics

	Data
Distance restraints	
Total NOE	6,136
Unambiguous	3,562
Ambiguous	2,574
H-bond Restraints	132
Total dihedral restraints	330
^3^J HNHA coupling constants	90
Total restraints	6,688
Structure statistics	
Violations	
rmsd of distance violation/constraint	0.022
rmsd of dihedral angle violation/constraint	0.294
rmsd from ideal geometry	
Bond length	0.002
Bond angles	0.510
Average pairwise rmsd	
Ordered backbone	0.9
All backbone	12.3
Ordered heavy atoms	1.3
All heavy atoms	12.3
Ramachandran statistics	
Most favored regions	98.4%
Allowed regions	1.6%
Disallowed regions	0%
Average Molprobity Clashscore	7.73
RCSB PDB ID	7UI5

In the hS100A9/M63F structure, Phe63 is in the same general orientation as Met63 in the wild-type structure (Fig. 3*C*). This leads, however, to a change in the relative orientations of the helices that make up the protein binding surface of S100A9, as follows: H2, H3a, H3b, and H4. In the crystal structure of the wild-type protein, a relatively large hydrophobic surface is exposed (red trapezoid, Fig. 3*E*). In contrast, this surface is significantly collapsed for hS100A9/M63F (Fig. 3*D*). This change is accompanied by an overall compaction of the structure (red dashed lines, Fig. 3 *D* and *E*). The full magnitude of this change is more easily seen with a video morphing between the two structures (*SI Appendix*, Video S1).

One way to quantify this change is by measuring the angle between H3b and H4. In the mutant NMR ensemble, this angle is 38.3° ± 2° (Fig. 3*D*). In the wild-type crystal structure (PDB 1irj), this is 68.0° (Fig. 3*E*). To put these numbers in context, we measured the angles between these helices for experimental structures of other members of the S100 protein family solved in both the absence and presence of calcium (shown in Fig. 2*A*). For apo structures, we found an angle of 16.7° ± 4° (*n* = 7); for calcium-loaded structures, we found an angle of 78.6° ± 23° (*n* = 25) (*SI Appendix*, Table S3). The hS100A9/M63F angle of 38.3° is almost exactly intermediate between the average apo and calcium angles. Thus, the M63F mutation stabilizes a conformation that has partially collapsed the hydrophobic protein-interaction patch, even while calcium is bound.

### Phe63 Stabilizes an Adjacent Helix.

We next sought to understand why this mutation causes this structural effect. To do so, we employed HDX measurements monitored by mass spectrometry (HDX-MS) (28, 29). In this experiment, the relative rates of deuterium uptake for backbone amides report on the local stability and solvent accessibility of those positions. HDX-MS monitors the exchange of amide hydrogens in the peptide backbone with solvent hydrogens (in this case, deuterium). An individual amide’s ability to undergo exchange is thus directly related to its solvent accessibility and local stability (30, 31). We monitored HDX on hS100A9 and hS100A9/M63F, quenching the reaction at various timepoints (lower pH and temperature). We digested each quenched sample using a combination of porcine pepsin and aspergillopepsin inline under acidic conditions and quantified the number of deuterium atoms per peptide by mass spectrometry (*SI Appendix*, Table S4). We then calculated the percentage of backbone amides that exchanged hydrogen for deuterium for each peptide at each timepoint. For details of the HDX experiment, see *Materials and Methods*.

**Fig. 4.**
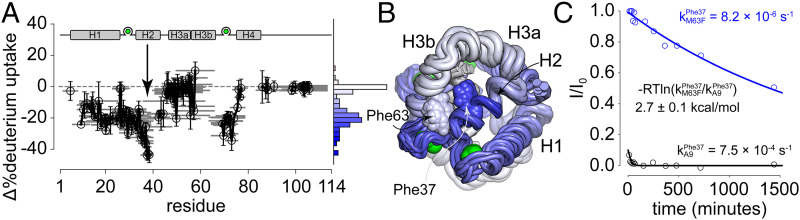
Phe63 stabilizes an adjacent helix. (*A*) Difference in peptide deuterium uptake observed by mass spectrometry between hS100A9 and hS100A9/M63F after 1 h. Gray lines indicate peptides observed. Circles indicate peptide centers. Errors represent uncertainty in difference in deuterium uptake for that peptide, as calculated by HDExaminer using default settings. Histogram on the right indicates frequency of peptides with differences in deuterium uptake, colored slowest (blue) to fastest (red) uptake. *SI Appendix*, Fig. S5 shows this in a larger format. (*B*) Uptake values from *A* mapped to the NMR structure of hS100A9/M63F using color and tube radius. To assign a value to each site, we took the average change in deuterium uptake for all peptides containing a given site. Colors correspond to the histogram shown in *A*. (*C*) Change in Phe37 backbone amide ^1^H/^15^N peak intensity when diluted into D_2_O. Lines show exponential decay model fit to the data. Colors denote protein hS100A9 (black) and hS100A9/M63F (blue).

Fig. 4*A* shows a plot of the difference in deuterium uptake for peptide fragments of hS100A9 and hS100A9/M63F after 1 h of exchange, where most peptides are in the transition and not yet fully exchanged. The M63F mutation causes a striking shift toward increased protection across the entire protein sequence. A total of 60% of peptides exhibit lower exchange in the mutant relative to the wild-type, while no position in the mutant exhibits statistically significant greater exchange than the wild-type (histogram in Fig. 4*A*). When mapped to the NMR structure (Fig. 4*B*), the HDX results point to increased stabilization of H1, H2, H3a, and H4. The most striking stabilization occurs between residues 30 and 39 in H2 (arrow, Fig. 4*A*). This region is directly adjacent to Phe63 in the NMR structure. Peptides containing Phe37, in particular, are highly stabilized in the mutant relative to the wild-type. In the NMR structure, the Phe37 sidechain forms an apparent edge-face π-stack with Phe63. This interaction bridges H2 and H3b and could conceivably be the proximate cause for the distorted orientation between these two helices.

We next tested the hypothesis that Phe37 is, indeed, stabilized by the M63F mutation. We repeated our HDX experiment using NMR, which provides site-specific rather than peptide-specific information. Using this approach, we were able to quantify differences in exchange rate at 25 °C for 25 matched amide backbones in hS100A9 and hS100A9/M63F (*SI Appendix*, Figs. S3 and S4 and Table S5). This includes five sites between residues 31 and 39 (,). All five sites exchanged between 60 and 600 times slower in the M63F mutant relative to hS100A9. As shown in Fig. 4*C*, the Phe37 backbone amide exchanges 92-fold slower in hS100A9/M63F (8.2 × 10^−6^ s^−1^) relative to hS100A9 (7.5 × 10^−4^ s^−1^). This corresponds to an apparent stabilization of 2.7 ± 0.1 kcal/mol. Thus, consistent with the HDX-MS experiments, our HDX-NMR experiments reveal that M63F strongly stabilizes Phe37 and other residues on H2.

### Phe37 and Phe63 Form a Strong Stabilizing Interaction.

Based on the NMR structure and our HDX data, we hypothesized that the Phe37/Phe63 interaction is the underlying cause of the stabilized, nonfunctional structure of the protein. We therefore constructed a mutant cycle measuring the effect of the Phe/Phe interaction on the stability and function of hS100A9. We constructed the cycle considering the evolutionary history of positions 63 and 37. The M63F mutation is a reversion to the ancestral state at position 63 (Fig. 1*B*). Over the same evolutionary interval, an ancestral Leu at position 37 evolved to Phe (12, 17). We therefore constructed a mutant cycle looking at all combinations of Leu/Phe at position 37 and Met/Phe at position 63.

We used the protein hS100A9/F37L as the reference state for this cycle. This protein does not have phenylalanine at positions 37 or 63 (L/M). By using it as our reference, we could measure the effects of introducing phenylalanine at position 37 (F/M; hS100A9), at position 63 (L/F; hS100A9/F37L/M63F), or at both positions (F/F; hS100A9/M63F). This allowed us to measure any nonadditive effect of the F/F pair on protein stability.

**Fig. 5.**
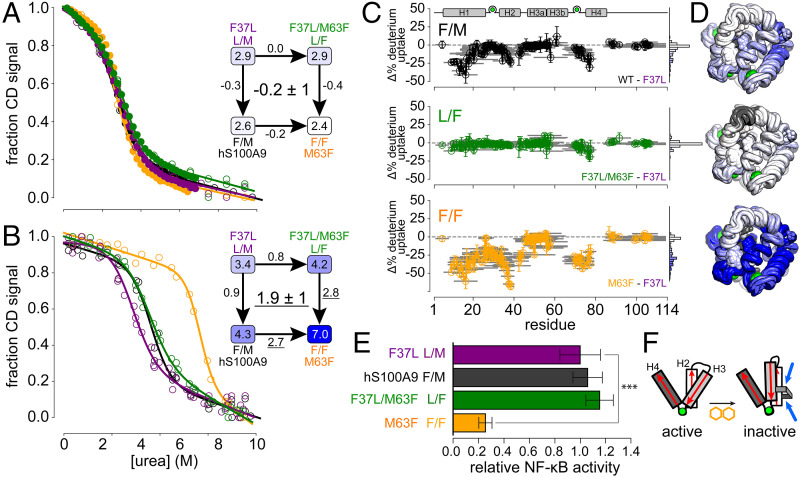
Phe37 and Phe63 form a stabilizing and functionally disruptive interaction. (*A*) Urea denaturation of S100A9 proteins in 0 mM Ca^2+^. Colors represent the variant hS100A9 (black), hS100A9/F37L (purple), hS100A9/M63F (orange), and hS100A9/F37L/M63F (green). Closed circles are automated titrations; open circles are manual titrations. Lines show best fit model to all experiments for each variant at this calcium condition. The *Inset* shows a mutant cycle based on the ΔG_unfold_ values extracted from fits shown (ΔG values in kcal/mol). Values along arrows are the effect of each mutation (ΔΔG_unfold_). The number in the *Center* indicates the coupling between Phe at each site. (*B*) Unfolding curves and mutant cycle for unfolding at 5 mM Ca^2+^. Visual elements are as in *A*. Stabilizing interactions noted in text are underlined. (*C*) Effects of Phe mutations on deuterium uptake after 1 h, calculated as the difference in the uptake of the indicated variant relative to hS100A9/F37L. Colors as in *A*. Graphical elements described in Fig. 4*A*. (*D*) Change in deuterium uptake mapped to the NMR structure of hS100A9/M63F for each variant in *C*. Colors follow histograms in *C*. (*E*) Protein variants (2 μM) activating an NF-κB luciferase reporter in HEK-293T cells transiently transfected with human TLR4, MD-2, CD14, and reporter plasmids. Bar lengths are the means of at least five biological replicates; error bars are the SEs on the mean. ***P < 0.001 (*t* test). Scale is normalized such that the activity of hS100A9/F37L is 1.0. (*F*) Schematic showing relative orientations of helices 2 to 4 for a single chain in an the active versus inactive structure. The aromatic/aromatic pair (yellow) forms a molecular staple (blue) that stabilizes the inactive conformation of the protein.

In the absence of calcium, we found that the stabilities of all four hS100A9 variants were indistinguishable within experimental uncertainty (Fig. 5*A*, ∼3 kcal/mol). As a result, there is no interaction between the M63F and L37F mutations outside experimental uncertainty (Fig. 5*A*, *Inset*, −0.2 ± 1 kcal/mol). With the addition of 5 mM Ca^2+^, a strong interaction emerged (Fig. 5*B*). Introducing L37F or M63F alone mildly increased stability (0.9 ± 1 and 0.8 ± 1 kcal/mol, respectively); introducing them together had a much larger effect (2.7 ± 1 and 2.8 ± 1 kcal/mol). This works out to a coupling energy of 1.9 ± 1 kcal/mol for the introduction of the new Phe37/Phe63 interaction (Fig. 5*B*, *Inset*; key energy terms underlined). All fit parameters are reported in *SI Appendix*, Table S1 (when we reanalyzed our results with individual m-values for each protein/calcium condition, we obtained an even higher coupling energy, namely, 4.8 ± 2 kcal/mol; *SI Appendix*, Fig. S6 and Table S2).

We next set out to validate the structural effect of the Phe/Phe interaction. We performed HDX-MS experiments on hS100A9/F37L and hS100A9/M63F/F37L and then calculated the change in deuterium uptake across sites relative to the hS100A9/F37L background. Fig. 5*C* shows the differences in uptake induced by F37 alone (black), F63 alone (green), or F37/F63 together (orange). When peptides were mapped to the structure, we found that they contained residues 30 to 39 (H2) and residues 70 to 80 (H4), which are massively more stabilized by the Phe/Phe pair than either Phe introduced alone (change in deuterium uptake, >20 to 70%; Fig. 5 *C* and *D*). As with the ΔG_unfold_ data, there is a strong synergistic stabilizing effect when F37 and F63 are introduced together.

### The Phe37/Phe63 Interaction Directly Disrupts Activity.

We next tested whether this interaction was responsible for the loss of proinflammatory activity in hS100A9/M63F. We measured the ability of the four hS100A9 variants to activate NF-κB via TLR4 in a luciferase reporter assay. We found that the variant with no Phe (L/M) and the variants with a single Phe (F/M and L/F) activated TLR4 indistinguishably from one another (Fig. 5*E*). In contrast, the F/F construct (hS100A9/M63F) had strongly compromised proinflammatory activity (Fig. 5*E*).

Our work shows that it is not Phe at position 63 that disrupts function but rather the combined effect of Phe at both positions 37 and 63. This leads us to the structural model shown in Fig. 5*F*; the interaction between Phe/Phe creates a new molecular staple between H3 and H2. This causes H3 to rotate relative to H4, thus distorting the structure of the molecule and disrupting proinflammatory function.

### Evolving S100A9 Proteins Avoid Having Phe at Both Position 37 and 63 Simultaneously.

**Fig. 6.**
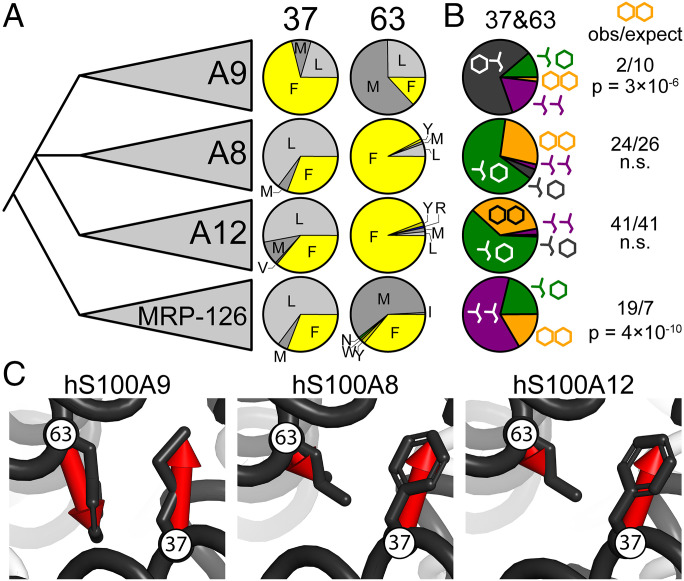
The Phe37/Phe63 state is rare in S100A9 proteins. (*A*) Frequencies of amino acids at sites 37 and 63 extracted from an alignment of 432 sequences containing 4 clades of S100 proteins (labeled on *Left*). Pie wedges are colored according to amino acid class, as follows: aromatic (yellow), nonaromatic/hydrophobic (gray), polar (green), or basic (blue). (*B*) Joint frequencies of nonaromatic and aromatic residues at positions 37 and 63 in each clade. Schematic Leu sidechain represents hydrophobic (Met, Leu, Val, or Ile in alignment); hexagon represents aromatic (Phe, Tyr, or Trp in alignment). Colors follow Fig. 5: nonaromatic/nonaromatic (purple), aromatic/nonaromatic (gray), nonaromatic/aromatic (green), and aromatic/aromatic (orange). Numbers to the *Right* indicate the observed and expected number of aromatic/aromatic pairs observed in that clade given the aromatic frequency at each site; *P* value is the result of a Fisher’s exact test. (*C*) Relative orientations of residues 37 and 63 in experimental structures of hS100A9 (PDB: 1IRJ) (18), hS100A8 (1MR8) (36), and hS100A12 (1E8A) (37). Red arrows connect the C_α_ and the most distal atom of the sidechain.

Having identified a pair of amino acids that, together, stabilize a nonfunctional form of the protein, we looked for evidence that this shaped the evolution of S100A9 orthologs. We constructed an alignment containing 432 protein sequences of S100A9 and its close paralogs from across amniotes (*SI Appendix*, Table S6). The proteins S100A9, S100A8, and S100A12 are found in mammals; MRP-126 is found in birds and reptiles (sauropsids). Excluding gaps, these proteins have sequence identities between 24% (human S100A8 vs. human S100A9) and 47% (human S100A9 vs. human S100A12). Although the relationship between these clades is difficult to establish with certainty, the most parsimonious scenario places MRP-126 as co-orthologous to S100A8, S100A9, and S100A12, which formed by serial duplication of the MRP-126 gene after the divergence of sauropsids and mammals (Fig. 6*A*) (12, 17).

Both positions 37 and 63 strongly prefer Leu, Met, or Phe to the exclusion of other amino acids in all four paralogs (Fig. 6*A*). S100A9 has noticeably different preferences than S100A8 or S100A12. Of the 107 S100A9 sequences in the alignment, 71% have Phe at position 37 and 13% have Phe at position 63. This preference is reversed relative to S100A8 and S100A12, which have ∼30% Phe at position 37 and ∼90% Phe at position 63 (Fig. 6*A*).

We next asked whether we saw patterns in the co-occurrence of Phe at positions 37 and 63 by measuring the frequencies of all four possible combinations of nonaromatic and aromatic residues at these two sites (Fig. 6*B*). This is directly analogous to our mutant cycles shown in Fig. 5. For S100A9, we found that aromatic/nonaromatic—e.g., Phe37/Met63—is the most common state. This is followed by nonaromatic/nonaromatic and nonaromatic/aromatic. Only 2 of the 107 sequences have aromatic/aromatic at both sites. If aromatic residues occurred independently at these sites, we would expect 10 sequences to have an aromatic/aromatic pair (71% × 13% × 107 sequences = 9.8). This depletion is statistically significant (*P* = 3 × 10^−6^; Fisher’s exact test). Thus, it appears that evolving S100A9 proteins can readily tolerate Phe at either position but avoid placing Phe at both positions simultaneously.

Such depletion is not observed for the other paralogs. S100A8 and S100A12 show no significant deviation in observed aromatic/aromatic pairs from what is expected given the frequency of aromatic residues at sites 37 and 63 (Fig. 6*B*). For MRP-126, we see relative enrichment of Phe/Phe pairs; we observe 19 pairs but only expect 7. Thus, it appears that the S100A9 clade has a unique evolutionary constraint to avoid placing Phe at both position 37 and 63 simultaneously. Given our experimental observations, a plausible constraint is avoiding the pathological stabilization of a nonfunctional conformation of the protein.

## Discussion

This work reveals how reverting a single residue to its ancestral amino acid in hS100A9 decreases its proinflammatory activity. The mutation creates a new stabilizing interaction between Phe37 and Phe63. This molecular staple reorients a helix, thus occluding the hydrophobic surface on S100A9 that is typically exposed upon calcium binding and thought to be critical for function (14, 15, 35). Having a Phe at either position modestly stabilizes S100A9 but has no impact on proinflammatory function, suggesting that generic stabilizing mutations in S100A9 do not affect its activity. Instead, we found that Phe can be functionally tolerated at either site individually but not at both sites together. This biochemical and functional constraint has left a detectable evolutionary footprint in the sequence preferences of evolving S100A9 proteins.

This evolutionary rule against Phe/Phe pairs appears to be specific to S100A9 but not other closely related proteins (Fig. 6*B*). Its closest paralogs—S100A8 and S100A12—exhibit no aversion for Phe/Phe. An earlier-diverging protein—MRP-126—even prefers Phe/Phe over other amino acid pairs at these positions. This differential preference for Phe/Phe can be understood by examining positions 37 and 63 in calcium-bound S100 protein structures. Fig. 6*C* shows the equivalent 37/63 positions in the structures of hS100A9, hS100A8, and hS100A12 (19, 36, 37). In hS100A9, these two residues are directly parallel and adjacent, allowing the formation of the Phe/Phe contact. In hS100A8 and hS100A12, these residues are slightly offset, presumably allowing Phe to be accommodated at both sites. While a structure of MRP-126 has not yet been solved, the observed preference for Phe/Phe in MRP-126 (Fig. 6*C*) indicates that these proteins can structurally accommodate both aromatic residues. This suggests that the MRP-126 structure might more closely resemble S100A8 and S100A12 structures at positions 37 and 63.

It is interesting to compare our results in which evolution avoids a specific interaction to another class of stabilizing residue pairs, namely, disulfide bonds. Overall, it appears disulfide bonds are generally under positive or neutral selection (32). Yet, this average almost certainly obscures specific instances in which disulfide bonds are deleterious (33). Likewise, Phe-Phe contacts are common features of proteins that are often critical for protein folding and stability; in the context of S100A9, however, the Phe-Phe contact is deleterious and thus avoided by evolution.

This change in structure and amino acid preference occurred on the same evolutionary interval over which S100A9 evolved potent proinflammatory activity relative to other S100 proteins (Fig. 1*B*) (12, 17). It is intriguing that S100A9 evolved enhanced activity along with this particular amino acid preference, whereas closely related S100s did not. Why do closely related S100s not select against Phe37-Phe63? We found, based on the structural analysis in Fig. 6, that amino acids at sites 37 and 63 do not physically interact outside the S100A9 clade, likely explaining the lack of evolutionary coupling between these sites for other S100 proteins. It is also possible that selection against this particular pair of residues is less important in other S100 proteins, as they do not activate TLR4 as potently as S100A9 (12, 17). It is unclear if the observed change in structure and amino acid preference (Fig. 6) was important for the acquisition of proinflammatory activity; however, once established, the new structure created a new constraint on sequence evolution; Phe is allowed at either site in S100A9s but not at both together. This is an example of evolutionary entrenchment (34, 38–42). The altered structure of S100A9 relative to its close paralogs makes a previously allowable Phe/Phe pair functionally deleterious, thus preventing reversion to the ancestral state of the protein.

This study provides further evidence that S100A9 activates inflammation by directly binding to TLR4 via the S100A9 hydrophobic patch exposed upon calcium binding. This hypothesis is consistent with the biology of the S100 protein family; S100s primarily engage with target proteins via calcium-induced exposure of a hydrophobic patch. We found previously that the full-length protein, not a proteolytic fragment, is the primary activator of TLR4 (12). Others found that mutating pairs of charged residues near the S100A9 hydrophobic patch (E64A/D65A, Q73A/E77A, E64A/E77A, D65A/Q73A) significantly reduces binding to TLR4/MD2 in vitro (16). Our in-depth analysis of the Phe37-Phe63 interaction is consonant with these findings; the Phe37-Phe63 interaction prevents S100A9/M63F from fully exposing the S100A9 hydrophobic patch upon calcium binding, resulting in drastically reduced S100A9 proinflammatory activity. The S100A9 binding site on TLR4 remains unknown; while an S100A9/TLR4/MD2 docking model has been proposed, this model is inconsistent with the structure of the (TLR4/MD2)_2_ heterotetramer bound by lipopolysaccharide (LPS), as S100A9 is predicted in the model to bind to the TLR4/TLR4 dimerization interface (16). Future studies will aim to directly determine the binding site and structure of S100A9 bound to the TLR4/MD2 complex to improve our understanding of S100A9-mediated inflammation.

We started this work asking what evolutionary constraints exist on stabilizing mutations. We can now answer this question for S100A9; the constraint at position 63 is not to avoid stabilizing the native conformation but rather to avoid stabilizing a nonnative conformation. In hS100A9, the most stable structure is the functional structure. In hS100A9/M63F, the most stable structure is a distorted, nonfunctional structure. A single interaction, between Phe37 and Phe63, is sufficient to stabilize this nonfunctional conformation. The fact that this can be achieved with a single mutation indicates that the nonfunctional conformation in wild-type S100A9 is only slightly less stable than the functional conformation in hS100A9—likely only a few kcal/mol given the apparent interaction energy between F37 and F63 (Figs. 4*C* and 5*B*).

From one perspective, the existence of an energetically similar but nonfunctional conformation is surprising. Selection for protein stability can lead to both positive design constraints (optimizing the native structure) and negative design constraints (deoptimizing nonnative structures) (43, 44). In an ideal case, evolution would find robust protein sequences for which no single mutation is sufficient to switch the protein from its native to a nonnative structure (44–48).

S100A9 may be less robust than expected because the protein undergoes a conformational change in response to calcium (Fig. 2*A*). The same protein sequence must therefore be compatible with two different structures. As a result of this degeneracy, the energy landscape for the protein may be interspersed with energetically similar, but nonfunctional, conformations (49–53). In this scenario, evolution cannot purge these structures from the energy landscape without disrupting the ability of the protein to respond to calcium.

Whatever their evolutionary origin, our work demonstrates that energetically similar, nonfunctional conformations constrain the evolution of protein sequences. Proteins exist as ensembles of structures, with some highly populated and others rarely seen (54–57). The same mutation can have different effects on different structures within this energetic ensemble, redistributing their relative populations (54, 55, 58). This means that proteins must not only avoid mutations that destabilize native structures but also avoid mutations that stabilize nonnative structures. As a result, we need to think of constraints on protein evolution in terms of multiple conformations—not simply in terms of the native conformation. Or, in the memorable words of Gregorio Weber, we should think of evolving proteins not as “platonic” single structures, but as “kicking and screaming stochastic molecules” (59).

## Materials and Methods

Raw data and scripts to perform all analyses and generate all figures are posted as a freely downloadable repository on github (https://github.com/harmslab/stability-constraint-ms-figures).

### Protein Expression and Purification.

We used a Cys-free variant of hS100A9 in a pETDuet-1 plasmid as our wild-type construct. This corresponds to Uniprot P06702 with a C3S mutation. We introduced the F37L and M63F mutations by site-directed mutagenesis (Agilent). We expressed the recombinant proteins in *Escherichia coli* BL21 (DE3) pLysS Rosetta cells grown in Luria broth (LB) containing both ampicillin and chloramphenicol. We streaked from glycerol stocks onto LB plates inoculated overnight cultures from single colonies and grew overnight cultures at 37 °C/250 rpm. The next morning, we inoculated 1.5 L of media with 10 mL of overnight culture and then allowed this to grow at 37 °C/250 rpm to an optical density at 600 nm (OD_600_) between 0.6 to 1.0. To produce isotopically labeled protein samples for NMR, we grew 2 × 1.5-L cultures to an OD_600_ between 0.6 and 1.0 as above. Cells were then pelleted at 3,000 rpm for 20 min, resuspended in 750 mL of 1× M9 minimal media, and grown at 37 °C/250 rpm for 45 min. M9 medium was prepared as follows: 1 L of 5× M9 stock solution was prepared by adding 30 g of NA_2_HPO_4_, 15 g of KH_2_PO_4_, and 2.5 g NaCl to 1 L of ddH_2_O, and then the mixture was autoclaved for 15 mins. A total of 750 mL of 1× M9 media for labeling was prepared by combining 150 mL of 5× M9 stock solution, 15 mL of 50% glucose (not included for ^13^C-labeled samples), 7.5 mL of Basal Medium Eagle Media (Thermo Fisher Scientific), 1.5 mL of 1 M MgSO_4_, 75 μL of 1M CaCl_2_, 0.75 g of ^15^NH_4_Cl (Sigma Aldrich) for ^15^N labeling (and 0.75 g of ^13^C glucose [Sigma Aldrich] for ^15^N/^13^C double labeling), and 750 μL of 1,000× antibiotic stocks (ampicillin and chloramphenicol), filled to 750 mL with ddH_2_O. We induced expression using 1 mM IPTG and allowed the cultures to grow overnight at 16 °C (or 4 h at 37 °C/250 rpm for isotopically labeled samples). Cells were then pelleted at 3,000 rpm for 20 min. After decanting the media, we stored the pellets at −20 °C. To lyse the cells, we resuspended the frozen pellets (3 to 5 g) in 25 mM Tris and 100 mM NaCl [pH 7.4], incubated the mixture for 20 min at 25 °C with DNase I and lysozyme (ThermoFisher Scientific), and then sonicated it on ice. We isolated the soluble fraction by centrifugation at 15,000 rpm for 20 to 60 min at 4 °C.

We purified the proteins using an Äkta PrimePlus fast protein liquid chromatography system. In its calcium bound form, hS100A9 naturally forms a high-affinity nickel binding site. We exploited this to purify the protein. We brought the soluble lysate to 2 mM CaCl_2_ and 25 mM imidazole and then ran it over a 5-mL HisTrap FF nickel-affinity column. We eluted the protein using a 50-mL gradient from 25 to 1,000 mM imidazole in 25 mM Tris, 100 mM NaCl, and 2 mM CaCl_2_ (pH 7.4). We pooled the peak elution fractions and dialyzed them overnight against 4 L of 25 mM Tris and 100 mM NaCl (pH 8.0) at 4 °C. We then ran the sample over a Q high-performance (HP) anion exchange column and eluted with a gradient of 100 to 1,000 mM NaCl. We checked for purity on an sodium dodecyl-sulfate polyacrylamide gel electrophoresis (SDS-PAGE) gel. If trace contaminants remained, we performed an additional Q HP anion exchange at pH 6, using the same base buffers and elution strategy as in the previous anion exchange step. All purified proteins were dialyzed overnight at 4 °C into 25 mM Tris and 100 mM NaCl (pH 7.4). We removed divalent ions by placing 2 g/L Chelex-100 resin (Biorad) into the dialysis container. We flash froze the proteins, dropwise, in liquid nitrogen and stored them at −80 °C. All proteins had a purity of >99% as assessed by SDS-PAGE.

### Biophysical and Biochemical Characterization.

For all experiments, we thawed protein aliquots on ice freezer stocks and then either dialyzed in the appropriate experimental buffer overnight at 4 °C or exchanged 3× into experimental buffer using 3K microsep spin concentrator columns (Pall Corporation). We filter sterilized all samples using 0.1-μm spin filters (EMD Millipore). Thawed aliquots were used for no more than 1 wk before they were discarded. We measured concentration by placing the protein into 6 M guanidinium HCl and measuring absorbance at 280 nm. We used an extinction coefficient of 6,990 M^−1^ cm^−1^, calculated using the ExPASy ProtParam tool.

### CD Spectroscopy and Chemical Denaturation Studies.

We performed equilibrium unfolding experiments using 2.5 μM S100A9 dimer, corresponding to 10 μM calcium binding sites. To equilibrate each protein sample fully at each measured urea concentration, we did at least one manual folding/refolding experiment where we pre-equilibrated samples in 0 or 9 M urea, mixed these samples at different ratios, and then allowed the mixtures to equilibrate overnight. We measured our final urea concentrations for each sample using their refractive index. For many variants, we complemented these manual titrations with automated titrations, in which we used an automatic titrator to gradually increase urea. For high calcium concentrations and some of the mutants, equilibration took longer than was feasible for an automated titration; therefore, we relied solely on manually titrations.

We did these experiments in 25 mM spectroscopy-grade Tris and 100 mM NaCl (pH 7.4). Manual unfolding and refolding curves were constructed by making concentrated 100 μM protein stocks in either buffer or 10 M urea at room temperature (25 °C) and then preparing dilutions at various concentrations of urea—causing the protein to either unfold or fold over equilibration. Samples were left to equilibrate in denaturant overnight at room temperature (>16 h). We measured the CD at 222 nm using a Jasco J-815 CD spectrometer. Unfolding/refolding equilibration was confirmed by comparing unfolded vs. refolded protein CD signal at the same concentration. CD signal was quantified at 222 nm in a 1-mm cuvette using a 1-nm bandwidth and standard sensitivity, and 2-s integration time. Detector voltage was < 600 V for all measurements.

We used lmfit (60) to fit a two-state unfolding model to the data to extract thermodynamic parameters (24)

$$\theta =b_{f}+m_{f}\left[\mathrm{urea}\right]+\left(b_{u}+m_{u}[\mathrm{urea}]\right)\frac{e^{-\left(\Delta G_{H2O}-m\left[\mathrm{urea}\right]\right)/RT}}{1+e^{-\left(\Delta G_{H2O}-m\left[\mathrm{urea}\right]\right)/RT}}$$

where θ is the fraction of molecules folded; *b_f_*, *m_f_*, *b_u_*, and *m_u_* are the folded and unfolded baseline y-intercepts and slopes; *ΔG_H2O_* is the unfolding free energy in water, *m* is the m-value; *R* = 0.001987 J⋅K^−1^⋅mol^−1^; and *T* = 298.15 K. We used two fitting strategies to analyze the data. We first allowed all six model parameters to float during regression for each protein/calcium condition. This yielded m-values ranging from 0.5 to 1.5, often yielding unrealistically curved native baselines (*SI Appendix*, Fig. S1 *B* and *C* and Table S2). To better constrain the fits, we next fit a single m-value across all variant/calcium conditions (*SI Appendix*, Fig. S1*A*). This yielded a global m-value of 0.95 kcal⋅mol⋅^−1^⋅M^−1^, with more consistent and reasonable native baselines for each protein/calcium condition (e.g., *SI Appendix*, Fig. S1 *B* and *C* vs. Fig. 2*C*).

It requires 11 more parameters to fit individual m-values to each experiment than to fit a single m-value to the entire dataset. We used an Akaike Information Criterion (AIC) test to ask whether the rmsd decrease for the individual fits was large enough to justify the extra parameters; the AIC test strongly favored the simpler, global m-value model (*P* = 0.00002). It is also reasonable to assume a single m-value for all four mutants, as the m-value is primarily determined by the amount of surface area buried in the hydrophobic core of the protein; this parameter is not expected to vary appreciably for these mutants. Finally, the ΔG values extracted by the two methods were qualitatively similar (*SI Appendix*, Fig. S1*D*), while the C_m_ values were almost identical (*SI Appendix*, Fig. S1*F*). The main text and display figures use the global m-value fit.

### NMR Experiments.

Samples were prepared in 25 mM Tris, 100 mM NaCl, 10 mM CaCl_2_ (pH 7.4), and 10% D_2_O. All protein concentrations ranged from 0.5 to 1.0 mM dimer. All NMR experiments were performed at 25 °C or 37 °C on an 800 MHz (18.8T) Bruker Avance IIIHD spectrometer at Oregon State University equipped with a triple resonance (HCN) cryoprobe. Two-dimensional (2D) ^15^*N*-TROSY spectra were collected with 32 transients, 1,024 direct points with a signal width of 12,820, and 256 indirect points with a signal width of 2,837 Hz in ^15^N. Backbone assignments were made based on the wild-type assignments (Biological Magnetic Resonance Databank identifier: 30017) (27) and confirmed using a suite of 3D experiments including HNCACB, HNCOCACB, HNCACO, and HNCO. Side chain assignments were made using 3D HCCH-TOCSY, 3D CCH-TOCSY, 2D HDCB, and 3D HECB. Distance restraints were obtained using a timeshared ^15^*N*-^13^C-NOESY-HSQC (61) with a mixing time of 160 ms. Three bond HN-HA coupling constants were measured with a 3D HNHA experiment. All 3D experiments were performed with nonuniform sampling percentages between 25% and 50%. All spectra were processed using NMRPipe (62); data were visualized using the CCPNMR or nmrviewJ analysis programs (63).

The NMR structure was determined with Xplor-NIH (64) using data from Talosn-derived dihedral angles, HN-HA 3-bond J-couplings, ^15^N and ^13^C NOE’s, and H-bonds inferred from secondary structure assignments. Nuclear Overhauser Effect (NOE) spectra were picked using automatic peak picking in nmrviewJ and obvious noise peaks removed manually. Preliminary structures were calculated using the PASD program from the Xplor-NIH software package, with initial probabilities set based on the crystal structure (PDB 1IRJ) (18). Structures were further refined with the final NOE assignments using Xplor-NIH. Additional distance restraints were included in the refinement stage to define the sidechain interactions with calcium based on the crystal structure.

For HDX experiments by NMR, protein samples were lyophilized and resuspended in 100% D_2_O immediately prior to measurement of 2D ^1^H-^15^N TROSY-HSQC spectra. Spectra were collected over 24 h, as follows: every ∼11 min for the first hour, every hour up to ∼8 h, and then a final spectrum after 24 h. Peak intensities were quantified using Sparky. We placed each residue into one of three classes, as follows: exchanged too quickly to quantify, exchanged too slowly to quantify, and exchanged on a timescale we could quantify. To make these classifications, we fit a two-parameter exponential decay model and a linear model with slope of zero to the peak intensity versus time for each residue. We used the AIC to determine select the model that best fit the data. Those residues for which we used the exponential model were quantifiable. Residues for which we used the linear model were classified as either too fast (relative intensity of <0.1) or too slow to quantify (*SI Appendix*, Figs. S3 and S4).

### HDX-MS Studies.

HDX-MS experiments were carried out using a LEAP PAL autosampler (LEAP Technologies). A total of 10 μM protein stocks were maintained at 4 °C. A total of 3 μL of a 10 μM protein sample was diluted into 27 μL of deuterated buffer at 20 °C (90% final deuterium concentration). After a varying labeling time, 27 μL of the labeled sample was quenched with 27 μL of 3.5 M GdmCl and 1.5 M glycine (pH 2.4) at 1 °C. A total of 45 μL of quenched sample was then immediately injected into an Thermo UltiMate 3000 ultra-high-performance liquid chromatography system (Buffer A, Buffer B). For inline digestion, samples were flowed over two immobilized protease columns (Upchurch C130B), namely, one with aspergillopepsin and one with porcine pepsin (conjugated to POROS 20 AL Aldehyde Activated Resin, Thermo Scientific), at a flow rate of 100 μL/min of buffer A. The digested protein was then desalted on a trap column (Upchurch C-128 with POROS R2 beads) for 2 min at a flow rate of 300 μL/min. An acetonitrile gradient (10% buffer B to 60% buffer B over 9 min) eluted peptides from this trap column onto an analytical C-8 column (Thermo 72205-050565) for separation before injection into an electrospray ionization source for mass spectrometry analysis on a Thermo Q Exactive mass spectrometer operating in positive ion mode.

For every protein variant, a nondeuterated control was subjected to the same liquid chromatography–mass spectrometry LC/MS protocol as the deuterated samples, and the resulting peptides were identified using tandem mass spectrometry (MS/MS). Peptide precursor spectra were acquired in data-dependent mode with the top 10 most abundant ions (charge state of ≥2, ≤6) selected for fragmentation and product ion analysis. Following MS/MS acquisition, precursor ions were excluded from further fragmentation for 4 s. The peptide identification software Byonic (Protein Metrics Inc.) was used to identify peptides from the MS/MS data. Following peptide identification, deuterium incorporation for each peptide at each labeling time was determined by centroid analysis with the software HDExaminer (Sierra Analytics). Data were not corrected for back exchange. All data shown are comparisons between different solution conditions collected on the same instrument. Previous back exchange controls with this experimental setup had an average of 22% back exchange (29).

### TLR4 Activity Assay and Cell Culture Conditions.

We purchased commercially distributed HEK293T cells from ATCC (CRL-11268). Because we used this cell line as a host for heterologous transient transfections, the appropriate control for consistency between assays was the measurement of reporter output for a set of control plasmids and a panel of known treatments. Upon thawing each batch of cells, we ran a positive control for ligand-induced response. We transfected the cells with plasmids encoding human CD14, human MD-2, human TLR4, Renilla luciferase behind a constitutive promoter, and Firefly luciferase behind an NF-κB promoter. We then characterized the raw luciferase output for five treatments, as follows: 1) mock, 2) LPS, 3) LPS + polymyxin B, 4) hS100A9 + polymyxin B, and 5) hS100A9 + 1.25x polymyxin B. LPS is an endotoxin positive control that activates TLR4. Polymixin B sequesters endotoxin present in the environment. This experiment has a stereotypical pattern of responses in Renilla luciferase (high for all) and Firefly luciferase (low, high, low, high, high). To ensure that the cells maintained their properties between passages, we repeated the mock, LPS, and LPS + polymyxin B control on every single experimental plate. This assay has a built-in control for mycoplasma contamination. A high Firefly luciferase signal in the absence of an added agonist indicates another source of NF-κB output in the cells—most plausibly, contamination. We discarded any cells that exhibited high background values or reached 30 passages.

All plasmids, cell culture conditions, and transfections for measuring the activity of S100s against TLR4s were identical to those previously described (12, 17, 65). Briefly, human embryonic kidney cells (HEK293T/17, ATCC CRL-11268) were maintained up to 30 passages in Dulbecco’s Modified Eagle Media (DMEM) supplemented with 10% fetal bovine serum at 37 °C with 5% CO_2_. *E. coli* K-12 LPS (LPS - tlrl-eklps, Invivogen) aliquots were prepared at 5 mg/mL in endotoxin-free water and stored at −20 °C. Working solutions were prepared at 10 μg/mL and stored at 4 °C to avoid freeze–thaw cycles. S100 proteins were prepared by exchanging into endotoxin-free phosphate-buffered saline (PBS) and incubating with an endotoxin removal column (Thermo Fisher Scientific) for 2 h. S100 LPS contamination was assessed by measuring activity with and without polymyxin B, an LPS chelating agent. Next, 2 μM (dimer) S100 treatments were prepared by diluting stocks of 25:75 endotoxin-free PBS:serum-free DMEM (Thermo Fisher Scientific). Polymyxin B (200 μg per 100 μL well) was added to limit activity due to background endotoxin contamination. Cells were incubated with treatments for 3 h prior to activity being assayed. We used the Dual-Glo Luciferase Assay System (Promega) to assay Firefly and Renilla luciferase activity of individual wells. Each NF-κB induction value shown represents the Firefly luciferase activity divided by the Renilla luciferase activity, background-subtracted using the LPS + polymyxin B activity for each TLR4 species and normalized to the activity of LPS alone for each TLR4 species to normalize between plates. All measurements were performed using three technical replicates per plate, a minimum of five biological replicates total, and a minimum of two separate protein preps.

### Sequence Curation and Alignment for Residue Coevolutionary Analysis.

We generated our alignment by BLASTing the human sequences (S100A8: P05109, S100A9: P07602, S100A12: P80511) and chicken sequence (MRP-126: P28318) against the National Center for Biotechnology Information nonredundant protein database (5,000 max hits; e-value cutoff of 0.001). We called the orthology of each BLAST hit by reciprocal BLAST against the combined human (GRCh38.p14) and chicken (bGalGal1.mat.broiler.GRCg7b) proteomes. We only included sequences that returned the S100A8, S100A9, S100A12, or MRP-126 as their top reciprocal BLAST hit. We manually checked the results of these orthology calls against our recently published phylogeny of this clade (17). This manual check was facilitated by highly distinct C termini for these four paralogs. We lowered redundancy in the dataset with an identity cutoff of 0.95. We aligned the sequences using Muscle5, followed by manual refinement. These analyses were done using topiary (https://github.com/harmslab/topiary) (69), which wraps Biopython (66) and Muscle5 (67). The final, edited alignment is available for download as a table (*SI Appendix*, Table S6) or fasta file.

## Supplementary Material

Supplementary File

Supplementary File

## Data Availability

Raw data and scripts to perform all analyses and generate all figures are posted as a freely downloadable repository on github (https://github.com/harmslab/stability-constraint-ms-figures) (70). The NMR structure of hS100A9/M63F has been deposited in the Research Collaboratory for Structural Bioinformatics (RCSB) Protein Data Bank with accession number 7UI5.
