# Supplementary material for: Evolution avoids a pathological stabilizing interaction in the immune protein S100A9
Source: Proc Natl Acad Sci U S A. 2022 Oct 4;119(41):e2208029119. doi: 10.1073/pnas.2208029119 (PMC9565474; doi:10.1073/pnas.2208029119)
Supplement: Supplementary File [file pnas.2208029119.sapp.pdf]

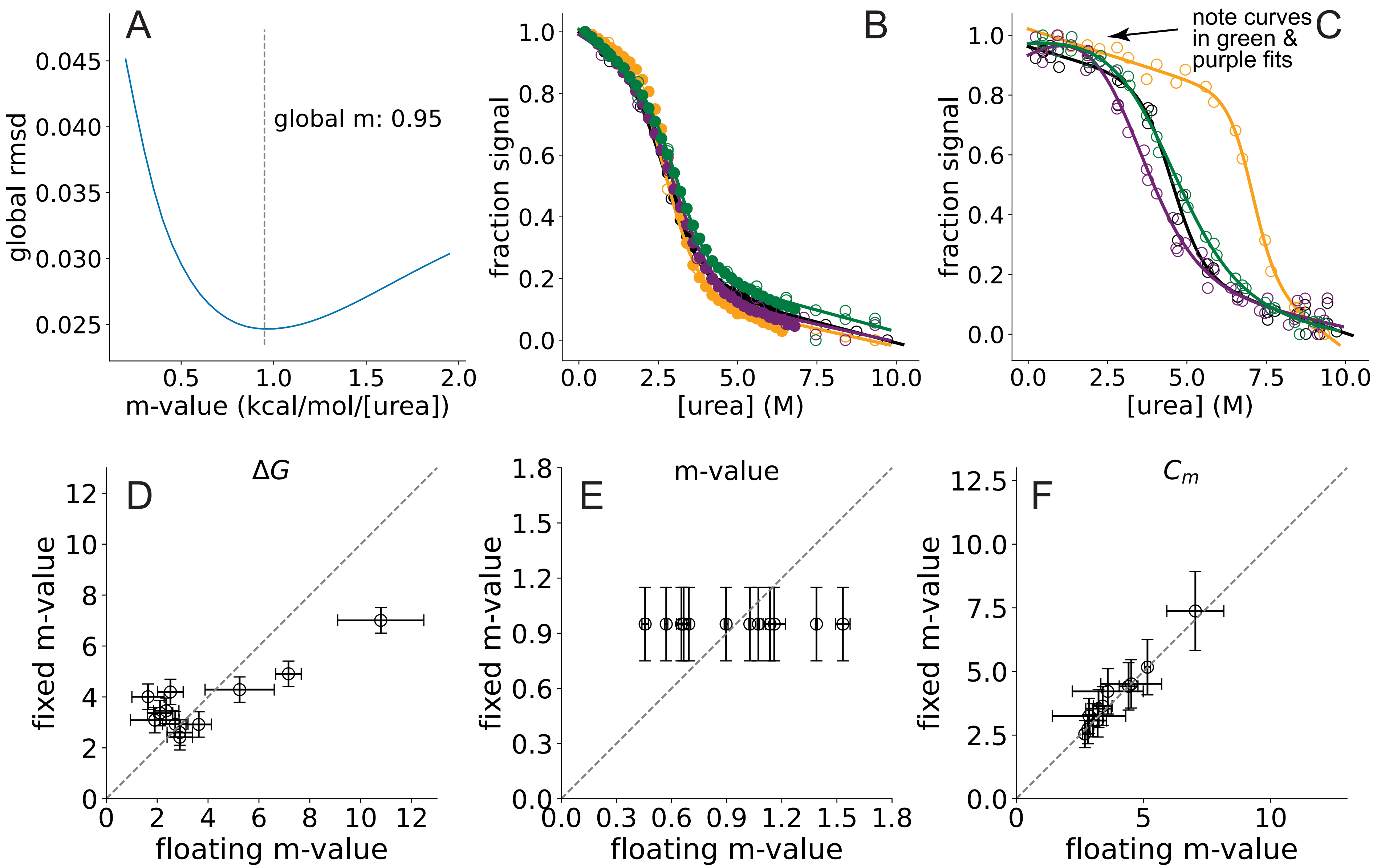

Figure S1: Comparison of individual and global m-value fit results. A) Global RMSD vs. m-value for simultaneous fits across all protein variants and calcium conditions. A value of 0.95 kcal/mol/M minimizes the RMSD. B and C) Unfolding curves at 0 mM and 5 mM CaCl2 fit with a floating m-value rather than a global m-value. (These curves correspond to those shown in Fig 5A and B). D) Comparison of estimated ΔG for each protein/calcium conditionfor a fixed m-value versus floating m-values. E) Comparison of the estimated m-value for each protein/calcium conditionfor a fixed m-value versus floating m-values. F) Comparison of the estimated Cm for each protein/calcium condition for a fixed m-value versus floating m-values.

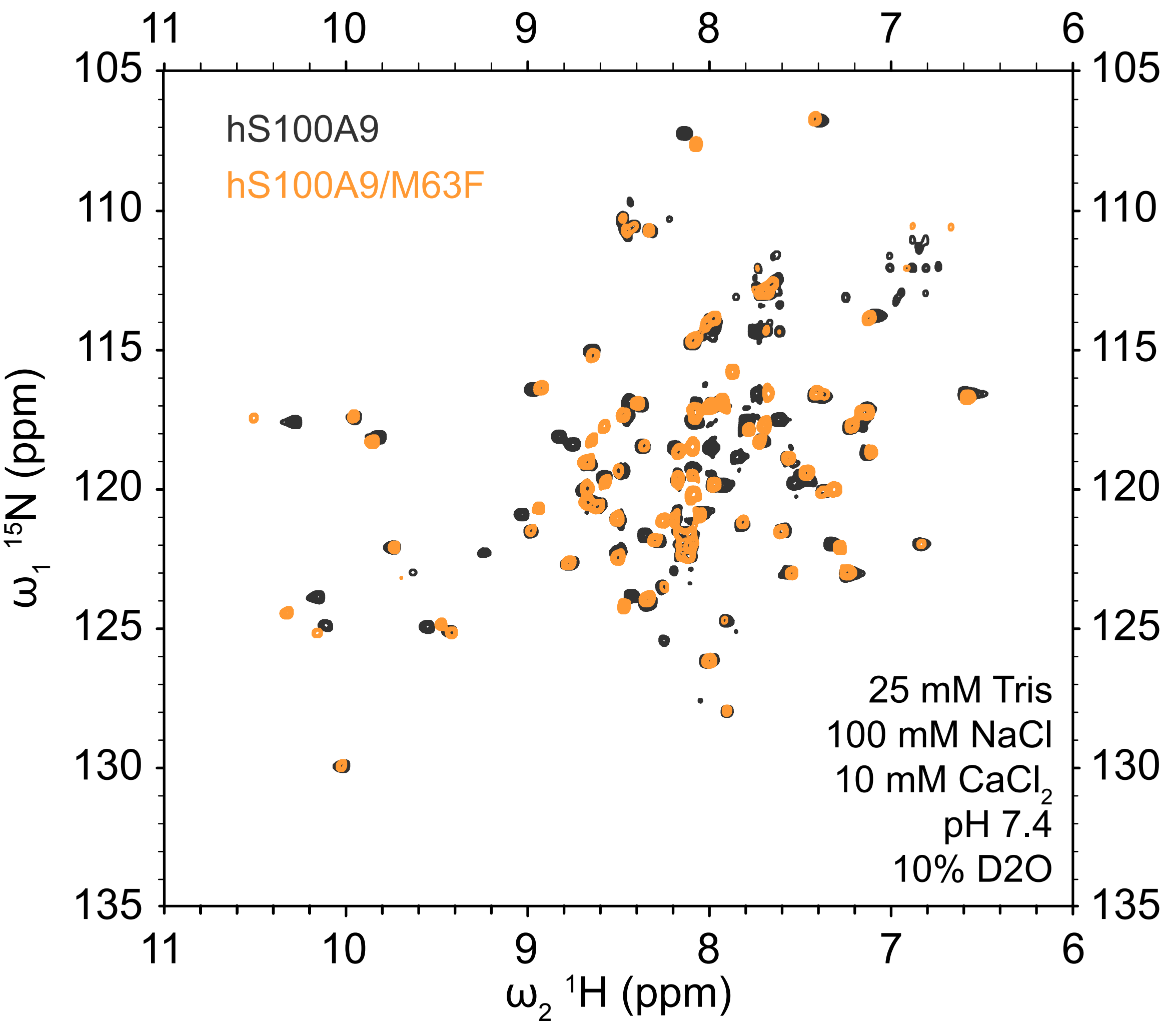

Figure S2: $^{1}H/^{15}N$ HSQC spectra of hS100A9 and hS100A9/M63F give well resolved peaks. HSQC spectra for 0.5-1.0 mM hS100A9 (black) and hS100A9/M63F (orange) under conditions indicated on the graph.

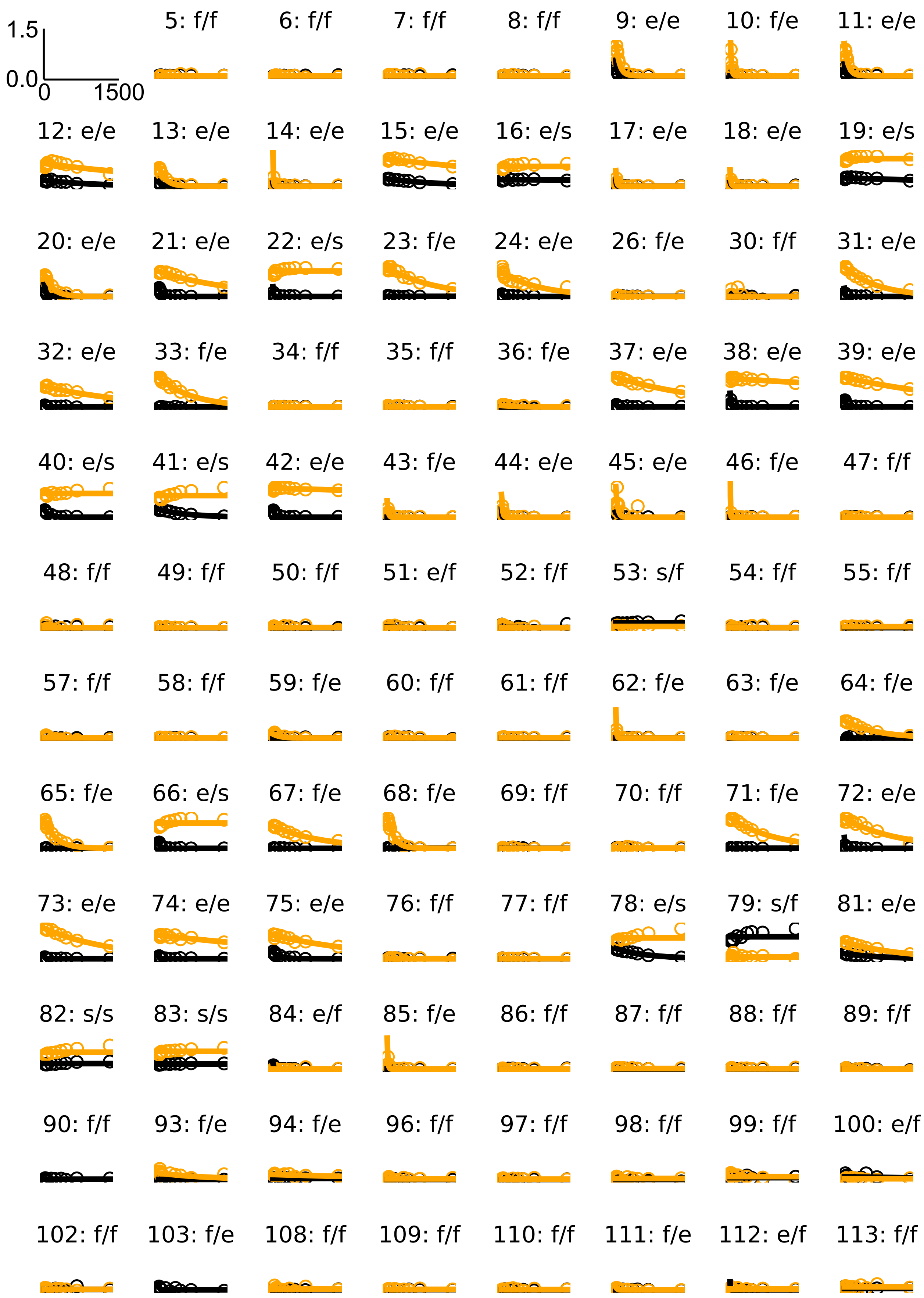

Figure S3: Individual amide backbone fits. Each subgraph is a residue (number indicated at top). Fit types are indicated by letter code: “e” (exponential fit), “f” (fast; exchange occurred too quickly for reliable fit); “s”: (slow, exchange occurred too slowly for reliable fit). x-axis is time (0-1500 min); y-axis is the peak intensity relative to intensity of the peak in an unexchanged protein (I/I0). Lines represent best fit to the data. Colors indicate hS100A9 (black) or hS100A9/M63F (orange).

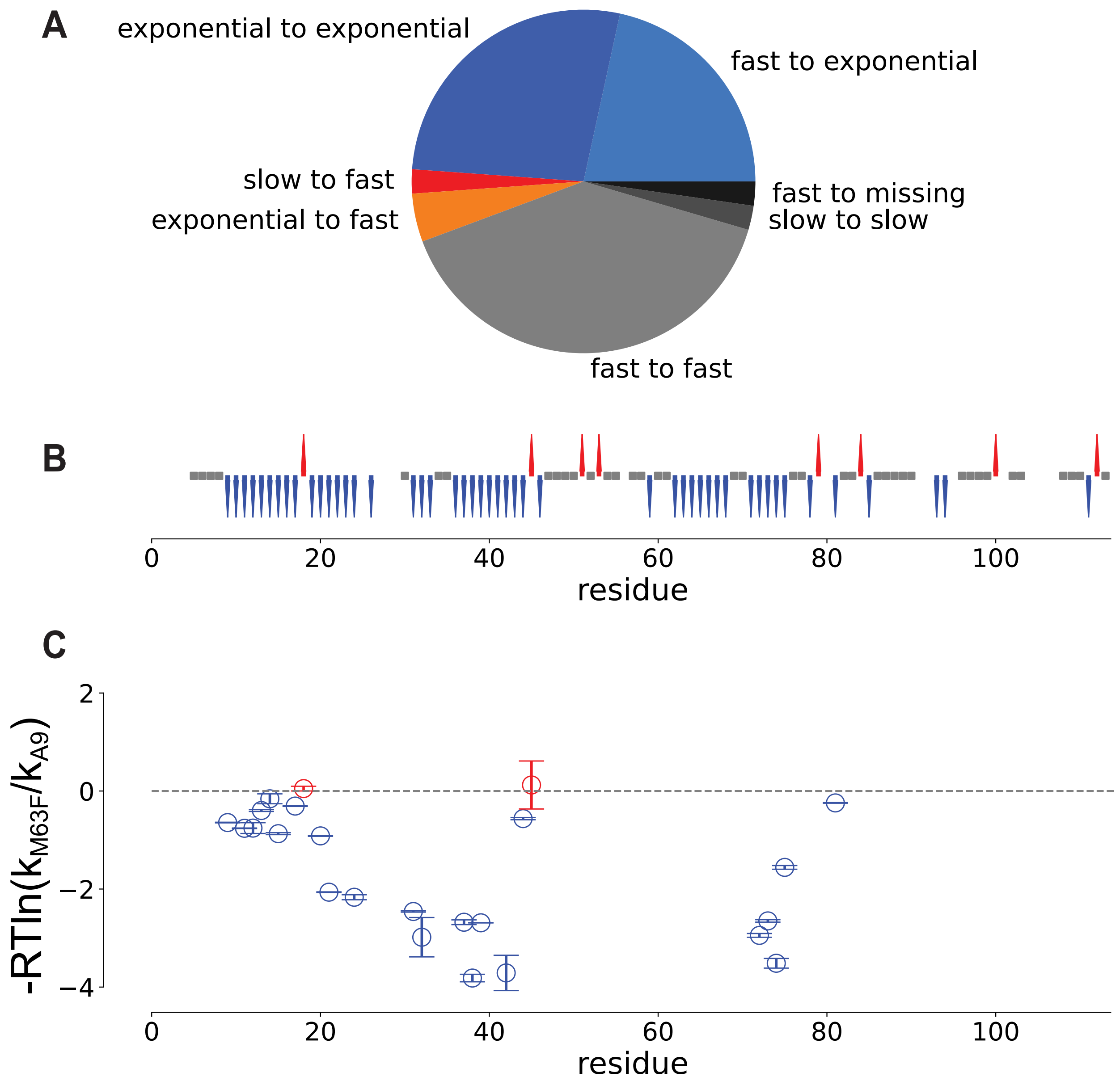

Figure S4: HDX measured by NMR identifies backbone amides in hS100A9/M63F stabilized relative to hS100A9. A) Pie chart summarizing fit types for residues under investigation. Text indicates change between hS100A9 and hS100A9/M63F. (For example, "fast to exponential" means the residue exchanged too quickly for a reliable fit in hS100A9, but had a reliable exponential in hS100A9/M63F). B) Qualitative changes in position stability. A blue arrow indicates sites more stable in hS100A9/M63F, either quantitatively ($k_{M63F} < k_{A9}$) or qualitatively (e.g. going from fast to exponential). Red arrows indicate sites more stable in hS100A9 using same criteria. Gray points indicate sites where the relative exchange rate of the sites could not be determined (e.g. both fast, both slow, or one missing). C) Relative rates of exchange for the 25 sites with exponential fits for both hS100A9 and hS100A9/M63F. Error bars are 95% confidence intervals on regression uncertainty. Colors indicate more stable in hS100A9/M63F (blue) or more stable in hS100A9 (red).

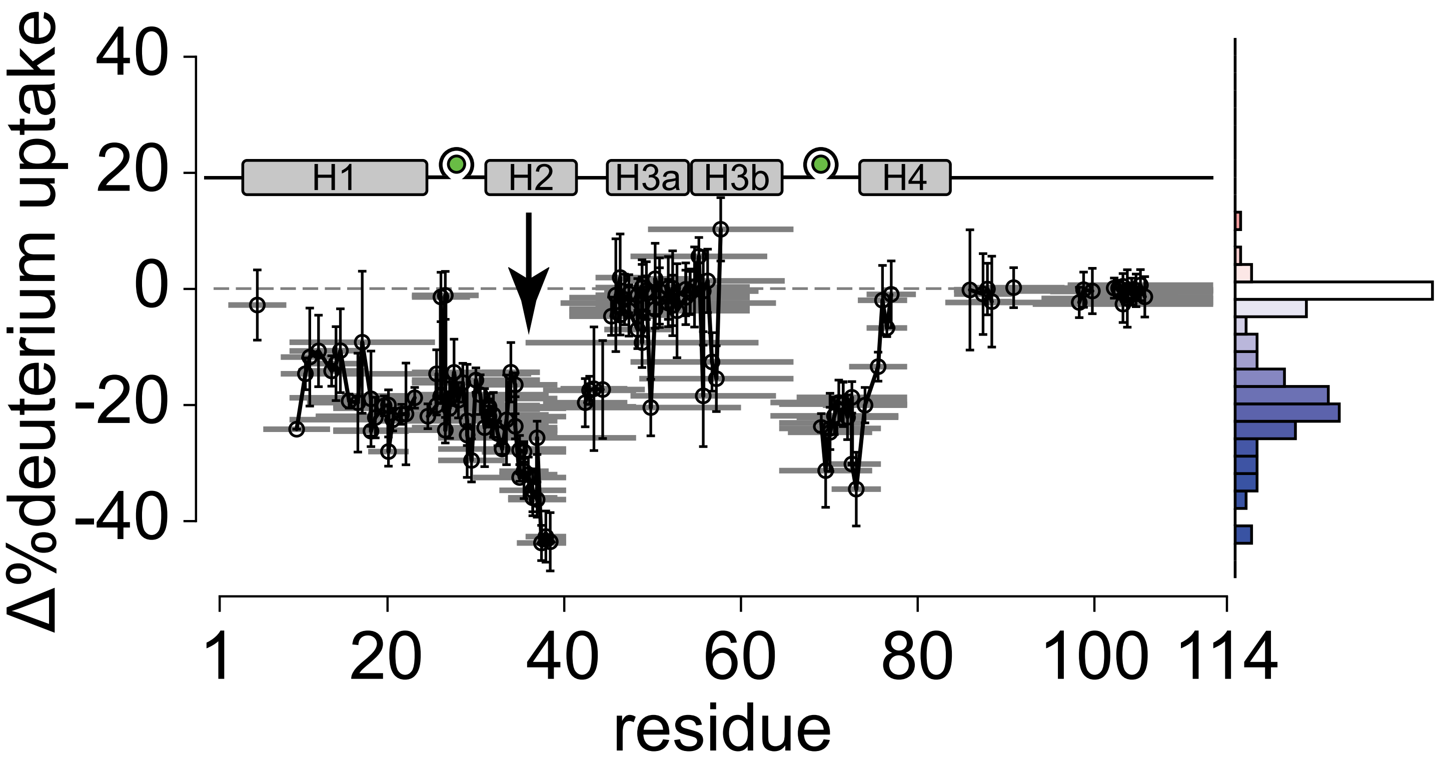

Fig S5: Difference in peptide deuterium uptake observed by mass spectrometry between hS100A9 and hS100A9/M63F after one hour. (Reproduces data shown in Fig 4A). Gray lines indicate peptides observed. Circles indicate peptide centers. Errors represent uncertainty in difference in deuterium uptake for that peptide, as calculated by HDExaminer using default settings. Histogram on right indicates frequency of peptides with differences in deuterium uptake, colored slowest (blue) to fastest (red) uptake.

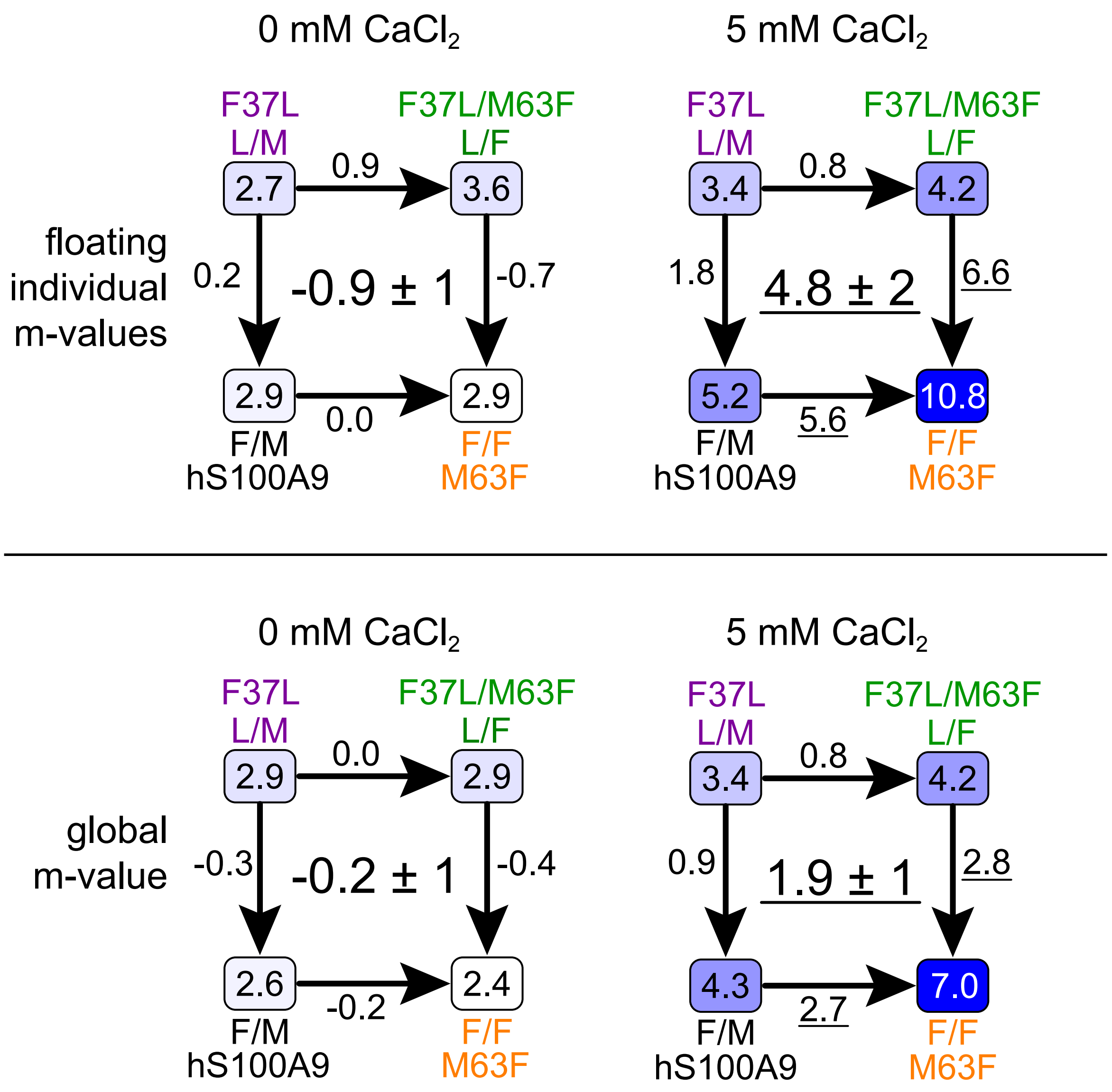

Figure S6: Calcium-dependent coupling between F37 and F63 is seen when unfolding curves are analyzed using either individual m-values or a global m-value. Top row: ΔG mutant cycles calculated from fits using individual m-values for reach protein/calcium condition. This corresponds to fits shown in Fig S1C,D, Table S2. Bottom row: ΔG mutant cycles calculated from fits using a global m-value (0.95 kcal/mol/M). This corresponds to fits shown in Fig 5A,B, Table S1).

Movie S1: The M63F mutation distorts the structure of S100A9. Morph between the wildtype hS100A9 structure (Figure 3E; PDB ID: 1IRJ) and the hS100A9/M63F structure (Figure 3D; this publication). Chains are traced as a tube. White and black correspond to the A and B chains of the homodimer. Movie starts with the hS100A9 structure, morphs to the hS100A9/M63F structure, then morphs back to the hS100A9 structure.
